# Wetlands in Changed Landscapes: The Influence of Habitat Transformation on the Physico-Chemistry of Temporary Depression Wetlands

**DOI:** 10.1371/journal.pone.0088935

**Published:** 2014-02-12

**Authors:** Matthew S. Bird, Jenny A. Day

**Affiliations:** 1 DST/NRF Research Chair in Shallow Water Ecosystems, Nelson Mandela Metropolitan University, Port Elizabeth, South Africa; 2 Freshwater Research, Department of Biological Sciences, University of Cape Town, Private Bag, Rondebosch, South Africa; Tennessee State University, United States of America

## Abstract

Temporary wetlands dominate the wet season landscape of temperate, semi-arid and arid regions, yet, other than their direct loss to development and agriculture, little information exists on how remaining wetlands have been altered by anthropogenic conversion of surrounding landscapes. This study investigates relationships between the extent and type of habitat transformation around temporary wetlands and their water column physico-chemical characteristics. A set of 90 isolated depression wetlands (seasonally inundated) occurring on coastal plains of the south-western Cape mediterranean-climate region of South Africa was sampled during the winter/spring wet season of 2007. Wetlands were sampled across habitat transformation gradients according to the areal cover of agriculture, urban development and alien invasive vegetation within 100 and 500 m radii of each wetland edge. We hypothesized that the principal drivers of physico-chemical conditions in these wetlands (e.g. soil properties, basin morphology) are altered by habitat transformation. Multivariate multiple regression analyses (distance-based Redundancy Analysis) indicated significant associations between wetland physico-chemistry and habitat transformation (overall transformation within 100 and 500 m, alien vegetation cover within 100 and 500 m, urban cover within 100 m); although for significant regressions the amount of variation explained was very low (range: ∼2 to ∼5.5%), relative to that explained by purely spatio-temporal factors (range: ∼35.5 to ∼43%). The nature of the relationships between each type of transformation in the landscape and individual physico-chemical variables in wetlands were further explored with univariate multiple regressions. Results suggest that conservation of relatively narrow (∼100 m) buffer strips around temporary wetlands is likely to be effective in the maintenance of natural conditions in terms of physico-chemical water quality.

## Introduction

Landscapes in human-populated regions have become intensively altered by anthropogenic activities and this landscape transformation has become a key driver of ecological systems worldwide (e.g. [1]–[4]). Information on the effects of terrestrial habitat transformation on wetland ecosystems is scarce, particularly so for small temporary wetlands, often a numerically dominant wetland type in seasonally dry areas [5], [6].

Physico-chemical constituents of the water column (e.g. pH, nutrients, conductivity) are regarded as potentially important determinants of biotic assemblage composition in wetlands and other freshwater ecosystems (e.g. [7]–[14]). More specifically, in the south-western Cape (South Africa) De Roeck [15] established that physico-chemical factors exert a significant structuring effect on invertebrate assemblage composition in temporary depression wetlands. Alteration of these factors through anthropogenic disturbance has potential to mediate ecosystem changes in these wetlands, through bottom-up effects on biota such as aquatic invertebrates and amphibians. Previous studies have focussed on permanent wetland types, from which various authors have reported significant effects of habitat transformation on an array of individual physico-chemical variables including turbidity, pH, nutrients, conductivity and dissolved oxygen [16]–[22]. Very few studies have specifically addressed relationships between terrestrial habitat transformation and physico-chemical conditions within temporary wetlands. Carrino-Kyker and Swanson [23] found a significant positive relationship between agricultural land use and conductivity levels in a study of thirty temporary pools in northern Ohio, USA. Brooks et al. [24] studied four ephemeral forest pools in Massachusetts, USA, and reported higher pH and conductivity, and lower concentrations of dissolved oxygen, for two of the pools occurring in urban areas compared with the two pools situated in undisturbed areas. Rhazi et al. [25] found higher levels of nutrients (nitrogen and phosphorus) in wetlands surrounded by agricultural fields than for those in natural areas for a set of ten temporary wetlands in Morocco. It appears that no universally consistent impacts of habitat transformation on physico-chemical conditions within temporary wetlands have been established thus far.

This study assesses the extent and nature of alterations to wetland physico-chemistry associated with human landscape transformation around temporary depression wetlands of the south-western Cape mediterranean-climate region of South Africa. Wetland physico-chemical characteristics are presented in relation to gradients of surrounding terrestrial habitat transformation induced by human activities. The term ‘habitat transformation’ is used hereafter with reference to the loss of natural terrestrial habitat around wetlands due directly (e.g. agriculture) or indirectly (e.g. alien invasive vegetation) to human land-use practices. A broad approach was taken by sampling a large number of wetlands across the south-western Cape region (n = 90). The survey design aimed to sample wetland sites across gradients of habitat transformation, defined in terms of the amount of surrounding habitat converted to agriculture, urban area, or invaded by alien vegetation. These are the three major agents of habitat transformation in the south-western Cape [26]–[28] and are all particularly prevalent on the low-lying coastal plains of the region [29], thus threatening temporary depression wetlands. The assessment of alien invasive vegetation as a type of habitat transformation around wetlands is a key element of the approach to this investigation. The negative effects of invasive vegetation on the quantity of groundwater available to aquatic systems in the region have been well documented [30]–[33], yet empirical studies that have addressed the influence of alien vegetation on surface water quality (e.g. physico-chemistry) of aquatic systems (lentic or lotic) are lacking. A single exception is the study of Bird et al. [34], who studied a set of 12 temporary depression wetlands within a Sand fynbos ecosystem in Cape Town, where the landscape has become differentially invaded by kikuyu grass, *Pennisetum clandestinum* Höchst. ex Chiov, and Port Jackson willow *Acacia saligna* (Labill) Wendl. They found that replacement of indigenous Sand fynbos habitat with alien vegetation results in lowered humic input to wetlands, with knock-on effects on other wetland physico-chemical constituents such as pH. The results of Bird and co-workers indicate the potential for broader effects on wetlands in the region, as a result of large-scale replacement of sclerophyllous fynbos vegetation with invasive plant species. This study investigates such broad-scale patterns, assessing whether the small-scale patterns observed by Bird and co-workers are also salient across the region.

Despite a paucity of information on the principal drivers of physico-chemical conditions in temporary depression wetlands, certain key factors have emerged from the literature, which include local geological substrate (soil properties), morphology of the wetland basin, surrounding landscape topography, surrounding terrestrial vegetation type and local climate (for reviews see [35]–[37]). Given that one or more of these driving factors were expected to be significantly altered by human habitat transformation (e.g. soil physico-chemical properties may be affected by the type of land use), we hypothesized that physico-chemical conditions in the studied temporary wetlands would in turn show significant association with changes in these driving variables and thus we expected physico-chemistry to be affected by surrounding habitat transformation. Specific relationships between each type of habitat transformation and each measured physico-chemical variable were explored to generate further hypotheses regarding effects of habitat transformation on wetland physico-chemistry in the region.

## Methods

### Ethics statement

Permission for fieldwork in the Agulhus National Park was granted by South African National Parks. A scientific collection permit was granted by Cape Nature, which allowed access to conservation areas under control of the provincial administration of the Western Cape and privately owned land in the province of the Western Cape. This research did not involve capture or handling of animals and therefore did not require approval of animal care and use procedures. The field study did not create effects on endangered or protected species.

### Study area

**Figure 1:**
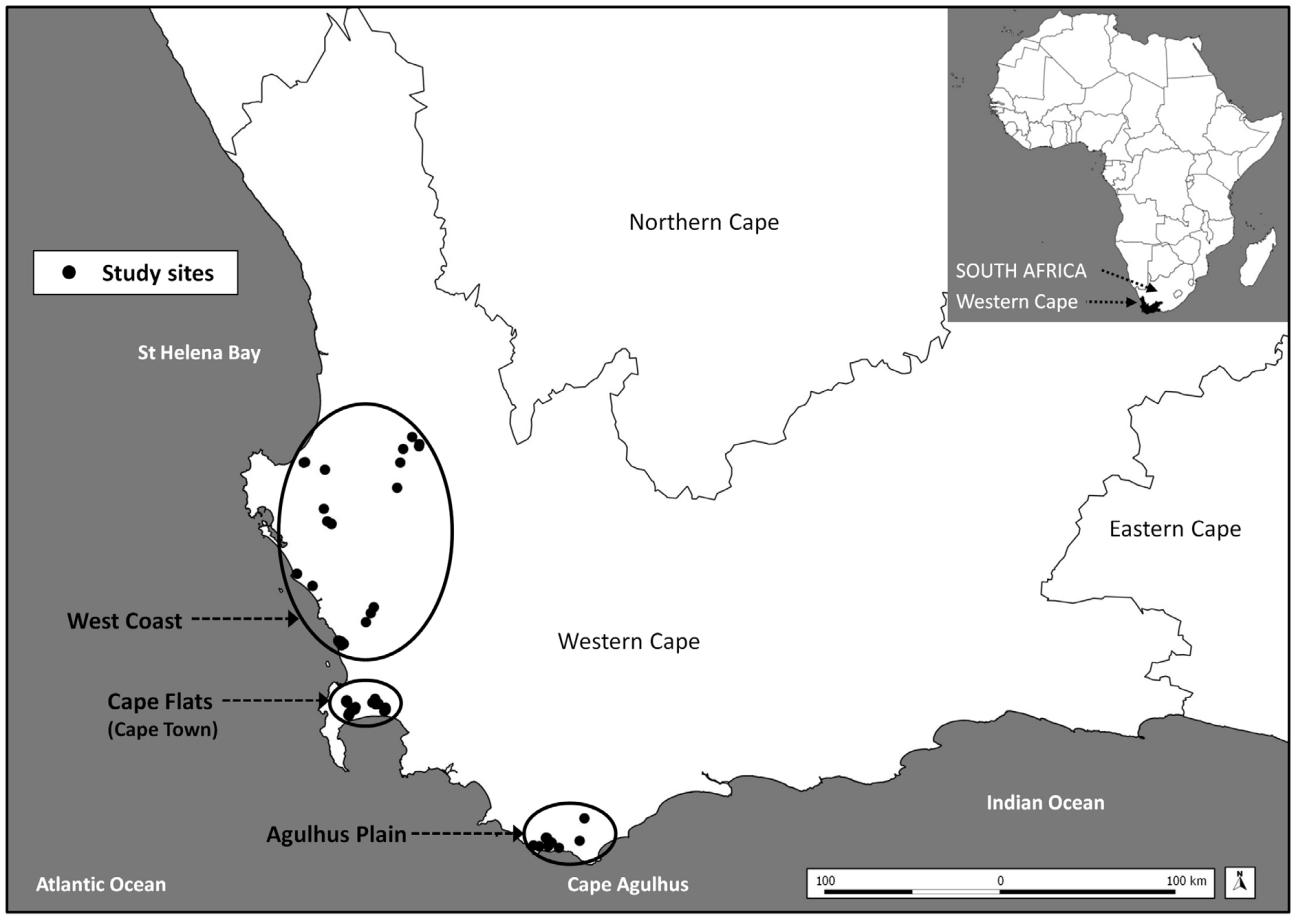
The south-western Cape study region showing sites sampled during the 2007 wet season (n = 90). Study sites were concentrated on three broadly distinguishable coastal plains (indicated by the bold circles). The region is bounded approximately by Cape Agulhus in the south and St Helena Bay in the north (modified from Bird et al. [39], for illustrative purposes only).

Ninety isolated depression wetlands (*sensu* Ewart-Smith et al. [38]) were sampled once during the winter-spring wet season (late-July to early-October) of 2007. The south-western Cape is unique in sub-Saharan Africa for having a mediterranean climate, typically encompassing cool, wet winters and warm, dry summers. The study area and selection of sites are described by Bird et al. [39] in a concurrent study on macroinvertebrate assemblages that utilised the same set of wetlands. Briefly, sampling covered an area from Cape Agulhus in the south to St Helena Bay in the north and incorporated three broadly distinguishable coastal plains (Figure 1). Wetlands were grouped *a priori* according to their natural (reference) state in terms of comparable soils and climate. Clusters of comparable wetlands were established using the vegetation groups of Rebelo et al. [40] as an indication of naturally comparable wetland groups, given the intimate link between vegetation type and local abiotic factors in the study region. In this regard, five wetland clusters were sampled in the study region (Appendix S1 in File S1), namely Sand fynbos (n = 44), Western strandveld (n = 28), Shale renosterveld (n = 6), Ferricrete fynbos (n = 6) and Sandstone fynbos (n = 6).

### Habitat transformation

The assessment of transformed habitat around each wetland was based on the quantification of the cover of natural vegetation (untransformed land), alien vegetation (predominantly *A. saligna* and *P. clandestinum*), agriculture and urban land within 100 and 500 m of each wetland [39]. An estimate of the areal cover of each habitat category was obtained from circular areas corresponding to 100 and 500 m radii from the edge of each wetland (i.e. approximately 0.03 and 0.8 km^2^). These scales were chosen because they could be accurately assessed on the ground without using GIS data. For both scales, the cover of each habitat type was estimated and assigned to one of four ordinal cover categories: 0 - none; 1 – sparse cover (<33%); 2 – moderate cover (33–66%); 3 – extensive cover (>66%). For those wetlands that were difficult to survey for a 500 m radius on foot, satellite imagery (Google Earth, accessed 2007) was combined with ground survey information, in order to score the ordinal categories of habitat cover. All estimates were made by the same person, in order to avoid inter-personal judgment biases. Appendix S2 in File S1 reports the habitat transformation scores for each wetland as raw data.

### Environmental variables

In order to record the range of physico-chemical conditions in each wetland, various biotopes were sampled. Biotopes were differentiated based on the structural complexity of habitats, of which four major types were encountered: complex vegetation (generally submerged, inter-woven, rooted or non-rooted with fine dissected leaves, including species such as *Isolepis rubicunda*, *Stuckenia pectinata*, *Chara glomerata* and *Paspalum vaginatum*); simple vegetation (typically rooted and emerging from the water surface, reed- or sedge-like vegetation, including species such as *Typha capensis*, *Phragmites australis*, *Bolboschoenus maritimus* and *Juncus kraussii*); open water (no vegetation, deeper than 30 cm); and benthic un-vegetated habitat (no vegetation, shallower than 30 cm). The percentage surface area covered by each of these four different biotopes in each wetland was recorded visually in the field. During field sampling it was noted that a maximum of three biotopes existed in any one wetland simultaneously. Thus, although all four biotope types were encountered among wetlands during field sampling, only three or fewer were represented within each wetland.

A number of *in situ* physico-chemical variables were measured in each of the biotopes within each wetland, producing three sets of *in situ* physico-chemical measures per wetland. For sites with only two biotopes, a double and a single set of physico-chemical readings were taken in the more and less abundant biotopes respectively. For sites where only one biotope covered the entire wetland, three replicate sets of physico-chemical readings were taken, with the aim of covering as much of the spatial extent of the wetland as possible among each set. All physico-chemical readings were taken at a standardized depth of 30 cm across all biotopes, with the exception of readings taken from habitats <30 cm deep. Measurements were taken as follows: pH using a Crison pH25 meter; dissolved oxygen with a Crison OXI45 oxygen meter; electrical conductivity (hereafter ‘conductivity’) with a Crison CM35 conductivity meter; and turbidity using a Hach 2100P turbidimeter. Temperature was recorded on the pH, oxygen and conductivity meters, although for analytical purposes an average of the readings across all three instruments was used.

Water column nutrient concentrations were measured at each wetland. Five 1L surface water samples were collected from each wetland, with the aim of covering the full spatial extent of each site, and pooled to form a bulk 5L sample. This was then thoroughly mixed and a 200 ml sub-sample was taken for analysis of nutrients levels in the laboratory. Samples were stored immediately in the dark at 4°C and upon return to the laboratory were frozen at −18°C. All samples were analysed within 30 days of collection from the field. NO_3_^−^+NO_2_^−^–N, PO_4_^3+^–P and NH_4_^+^–N concentrations were estimated using a Lachat Flow Injection Analyser. Approximate detection limits are: for PO_4_^3+^ 15 µg.L^−1^ P; for NO_3_^−^ and NO_2_^−^ 2.5 µg.L^−1^ N; and for NH_4_^+^ 5 µg.L^−1^ N. These variables are hereafter referred to in the text as ‘phosphates’, ‘nitrates + nitrites’ and ‘ammonium’ respectively. The geographical position and altitude at the centre point of each wetland were recorded using a Garmin eTrex Vista handheld GPS device (point accuracy of 3 m). In order to make sure no permanently inundated wetlands were included in the dataset, only sites with maximum depth <2 m were sampled. Most of the deeper sites were re-visited in summer to confirm that they had dried up. Appendix S3 in File S1 reports the raw environmental data collected at each wetland.

### Data analysis

Separate subsets of the dataset were used to analyse relationships between each type of habitat transformation and physico-chemical conditions in wetlands. These subsets were composed of sites affected by only one type of habitat transformation (e.g. agriculture). This was done to exclude sites that were affected by habitat transformations other than the type of interest. Each separate dataset was composed of least impaired sites (surrounded by extensive indigenous vegetation) and those sites that were impacted by varying degrees of habitat conversion for the given transformation type. Only least impaired sites occurring within the same wetland cluster as impacted sites were selected, to ensure comparison with naturally similar wetlands in the area. Two different but largely overlapping datasets were created for each habitat transformation type, corresponding to the 100 and 500 m scales of analysis, because in certain cases sites used in analysis of impact at one scale were not applicable at the other. An exception to this was for analyses relating to the amount of natural (indigenous) vegetation cover around wetlands, as this criterion was applicable to all cases.

Two broad types of response data were analysed in relation to surrounding gradients of habitat transformation, namely physico-chemical variables as a set (multivariate response) and individual physico-chemical variables (univariate response). Table 1 provides a list of the variables analysed in this study. The names used for these variables in Table 1 are hereafter used in the text. For multivariate analyses, response variables were first normalized and then converted into a Euclidean distance matrix, which was subsequently related to surrounding levels of habitat transformation. For the physico-chemical variables that were measured *in situ* for each biotope, an average of the three readings per wetland was used in subsequent analyses. Multivariate linear regressions were used to relate the physico-chemical response matrices to the habitat transformation predictor variables. Following the recommendations of Somerfield et al. [41], the gradient analyses in this study are best performed using regression rather than ANOVA techniques, given that the predictor variables representing gradients of impact are ordinal. Detrended Correspondence Analysis (DCA) indicated that gradient lengths in the physico-chemical dataset were best suited to linear rather than unimodal analyses [42]. Multivariate regressions were performed using distance-based Redundancy Analysis (dbRDA, [43], [44]), a non-parametric multivariate regression procedure based on any given dissimilarity measure, in this case Euclidean distance. Patterns in the multivariate physico-chemical data were visually explored using Principal Components Analysis (PCA) ordination. Sites were coded on each PCA plot according to three factors of interest, namely surrounding overall levels of habitat transformation (‘Natural 100 m’ and ‘Natural 500 m’, Table 1), the wetland cluster into which they were classified (defined by vegetation type, Table 1), and at a broader level, the coastal plain on which they were situated (West Coast, Cape Flats and Agulhus Plain, Figure 1). These factors were incorporated in order to assess the variation in physico-chemical conditions among wetlands in relation to habitat transformation gradients, as well as natural spatial factors.

**Table 1:** List of the physico-chemical response variables, habitat transformation predictor variables and spatio-temporal covariables incorporated into the analyses of this study.

Variable type	Variable scale	Category/set	Variable name	Description
Response variables	Quantitative (continuous)	Physico-chemistry		
			pH	Measured *in situ* for each biotope
			Conductivity	Measured *in situ* for each biotope
			Temperature	Measured *in situ* for each biotope
			Turbidity	Measured *in situ* for each biotope
			Dissolved oxygen	Measured *in situ* for each biotope
			Nitrates + nitrites	Integrated sample from across the wetland
			Phosphates	Integrated sample from across the wetland
			Ammonium	Integrated sample from across the wetland
Predictor variables	Semi-quantitative (ordinal)	Habitat transformation		
			Natural 100 m	Areal cover of indigenous vegetation within 100 m radius of wetland edge
			Natural 500 m	Areal cover of indigenous vegetation within 500 m radius of wetland edge
			Invaded 100 m	Areal cover of alien invasive vegetation within 100 m radius of wetland edge
			Invaded 500 m	Areal cover of alien invasive vegetation within 500 m radius of wetland edge
			Agriculture 100 m	Areal cover of agriculture within 100 m radius of wetland edge
			Agriculture 500 m	Areal cover of agriculture within 500 m radius of wetland edge
			Urban 100 m	Areal cover of urban surface within 100 m radius of wetland edge
			Urban 500 m	Areal cover of urban surface within 500 m radius of wetland edge
Covariables	Quantitative (continuous)	Spatio-temporal		
			Longitude	Taken at the wetland centre-point
			Latitude	Taken at the wetland centre-point
			Altitude	Taken at the wetland centre-point
			Time	Number of days since first sampling event
	Categorical	Spatio-temporal		
			Ferricrete fynbos	*Indigenous terrestrial vegetation type
			Sand fynbos	*Indigenous terrestrial vegetation type
			Sandstone fynbos	*Indigenous terrestrial vegetation type
			Shale renosterveld	*Indigenous terrestrial vegetation type
			Western strandveld	*Indigenous terrestrial vegetation type

* Either presently or historically surrounding each wetland (vegetation types s*ensu* Rebelo et al. [40]).

Univariate multiple linear regression models were used to test for relationships between individual physico-chemical variables and gradients of habitat transformation. The coefficient of partial determination (partial r^2^) was incorporated into the univariate regression results by squaring the partial correlation coefficient (r) for the predictor variable of interest [45]. Univariate relationships were visualized using partial residual plots (*sensu* Larsen & McCleary [46]), which allow one to hold the covariables constant in each model and also allow visual examination for heterogeneity in the spread of residuals, deviations from linearity and outliers [45], [47]. Potential outliers were quantitatively assessed using Cook's distances (Cook's D_i_, *sensu* Cook and Weisberg [48]), where D_i_ values >1 or D_i_ values considerably larger than the rest of the values would warrant an outlier [45]. All univariate and multivariate regression models were conditioned upon covariables, so as to partial out the effects of potentially confounding factors. Covariables included the following measures for each wetland: longitude and latitude (decimal degrees); time (days since first sampling event); altitude (m); and vegetation type (five dummy variables defined the wetland clusters). Where there was urban land surrounding wetlands, there was often invasive vegetation and *vice versa*. To help address this overlap, when assessing relationships between invasive vegetation cover and wetland physico-chemical conditions, the amount of urban land cover was specified as a covariable, and *vice versa* when assessing the effects of urban land cover as the primary variable. To maximise parsimony, covariable subsets were pre-selected for each model using step-wise regression of each response variable or matrix on the full list of possible covariables (Table 1, Appendix S1 in File S1).

Environmental variables were log_10_ transformed where appropriate, in order to achieve normality. A standard α level of 0.05 was used to assess the statistical significance of regression relationships, except for tests related to agriculture, as the smaller sample size of the two agricultural datasets (100 m: n = 24; 500 m: n = 21) indicated that the possible lack of power to detect effects could be countered by interpreting P values <0.10 as offering some evidence against the null hypothesis. Due to the possibility of inflated Type 1 error for multiple testing the significance of regressions was also assessed at a more conservative α level of 0.01, as well as after sequential Bonferroni correction (*sensu* Holm [49]). The Bonferroni procedure is likely to yield inappropriately conservative results (inflated Type 2 error) for univariate testing in this study, given the large number of tests conducted. Significant results (for both α<0.05 and α<0.01) were interpreted with caution if there were only one or two significant variables out of a large group of tested variables [50], [51]. DCA ordinations were performed using CANOCO for Windows v4.5 [52]. All dbRDA models (including the step-wise models) were implemented using the DISTLM routine of the PERMANOVA+ package [53]. P values for dbRDA models were tested by 9999 permutations of residuals under the reduced model. PCA ordinations were performed using PRIMER v6 software [54], [55]. Univariate multiple regressions (including step-wise models and partial residual plots) were performed using STATISTICA v10 software (Statsoft Inc. 2010, Tulsa, Oklahoma, USA).

## Results

### Physico-chemical characteristics of wetlands

Wetlands generally had a neutral pH or were slightly alkaline, although several highly acidic sites were encountered in the Sand fynbos cluster and several highly alkaline sites were spread among the clusters (for pH ranges per wetland cluster see Appendix S4 in File S1). Conductivity levels (as a proxy for salinity) were generally low, with mean and median values per wetland cluster all below 5 mS.cm^−1^. Turbidity levels were low on the whole and had mean and median values across all wetland clusters <10 NTU, except for the Shale renosterveld cluster, which stood out for having higher values (reflecting the naturally high quantity of clay particles in these shale-derived soils). Dissolved oxygen concentrations varied between moderate and high levels (range: 4.05–9.85 mg.L^−1^) in terms of mean and median values among the wetland clusters. Mean and median nutrient concentrations were low, except for the high values reported for phosphates and ammonium in Shale renosterveld wetlands (phosphates range: 12.03–999.41 µg.L^−1^; ammonium range: 61.72–2803.87 µg.L^−1^). Several extremely nutrient-enriched sites were found in the Sand fynbos cluster, as reflected by the very high maximum values for all three nutrient variables in this cluster (nitrates + nitrites maximum: 8241.59 µg.L^−1^; phosphates maximum: 2827.36 µg.L^−1^; ammonium maximum: 4231.53 µg.L^−1^, Appendix S4 in File S1).

### Multivariate patterns

Physico-chemical conditions in wetlands were significantly related (α<0.01 and also after Bonferroni correction) to the cover of natural (indigenous) vegetation within 100 m (P = 0.002) and 500 m (P = 0.009), and the cover of alien invasive vegetation within 100 m (P = 0.005) and 500 m (P = 0.005) (Table 2). Wetland physico-chemistry and urban cover within 100 m of wetlands were significantly related at α<0.05 only (P = 0.022). Despite some of the results being significant, very little of the variation in physico-chemical conditions was explained by these land cover variables (range: 2.08–5.57%) in comparison to that explained by the spatio-temporal covariables (range: 35.57–43.09%). No significant relationships were found between physico-chemical conditions in wetlands and urban cover within 500 m or agricultural cover within 100 and 500 m (Table 2).

**Table 2:** Non-parametric multivariate regression tests (dbRDA) for relationships between habitat transformation gradients and physico-chemical conditions in wetlands.

Predictor variable	Res. df	F	P	% Var	Covariables	% Var (covariables)
Natural 100 m	82	3.962	0.002***	2.62	Time, longitude, latitude, altitude, SF, SR	43.09
Natural 500 m	82	3.106	0.009***	2.08	Time, longitude, latitude, altitude, SF, SR	43.09
Invaded 100 m	65	3.529	0.005***	3.26	Time, longitude, latitude, altitude	36.71
Invaded 500 m	66	3.441	0.005***	3.18	Time, longitude, latitude, altitude	35.79
Agriculture 100 m	18	1.333	0.243	2.50	Time, longitude, latitude, SR	63.71
Agriculture 500 m	16	1.186	0.299	3.45	Time, FF, SR	50.04
Urban 100 m	31	2.927	0.022*	5.57	Time, longitude, latitude, altitude	35.47
Urban 500 m	49	1.879	0.090	2.24	Time, latitude, altitude, Invaded 500 m	39.37

Natural - indigenous vegetation; Invaded - alien invasive vegetation; Agriculture - agricultural land; Urban - urban area. The areal cover of these variables is represented within 100 and 500 m radii of each wetland edge. To maximise parsimony, covariable subsets were pre-selected for each model using step-wise regression of each response matrix on the full list of possible covariables (see Table 1). % Var - the percentage of variation in each Euclidean distance matrix (normalized physico-chemical variables) that is explained by each respective predictor variable or covariable set in each model; Time – number of days since the first sampling event; SF – Sand fynbos; SR – Shale renosterveld; FF – Ferricrete fynbos; Res. df – residual degrees of freedom for each model. Significant P values at α<0.05 (*), α<0.01 (**) and after sequential Bonferroni correction (***) are indicated.

**Figure 2:**
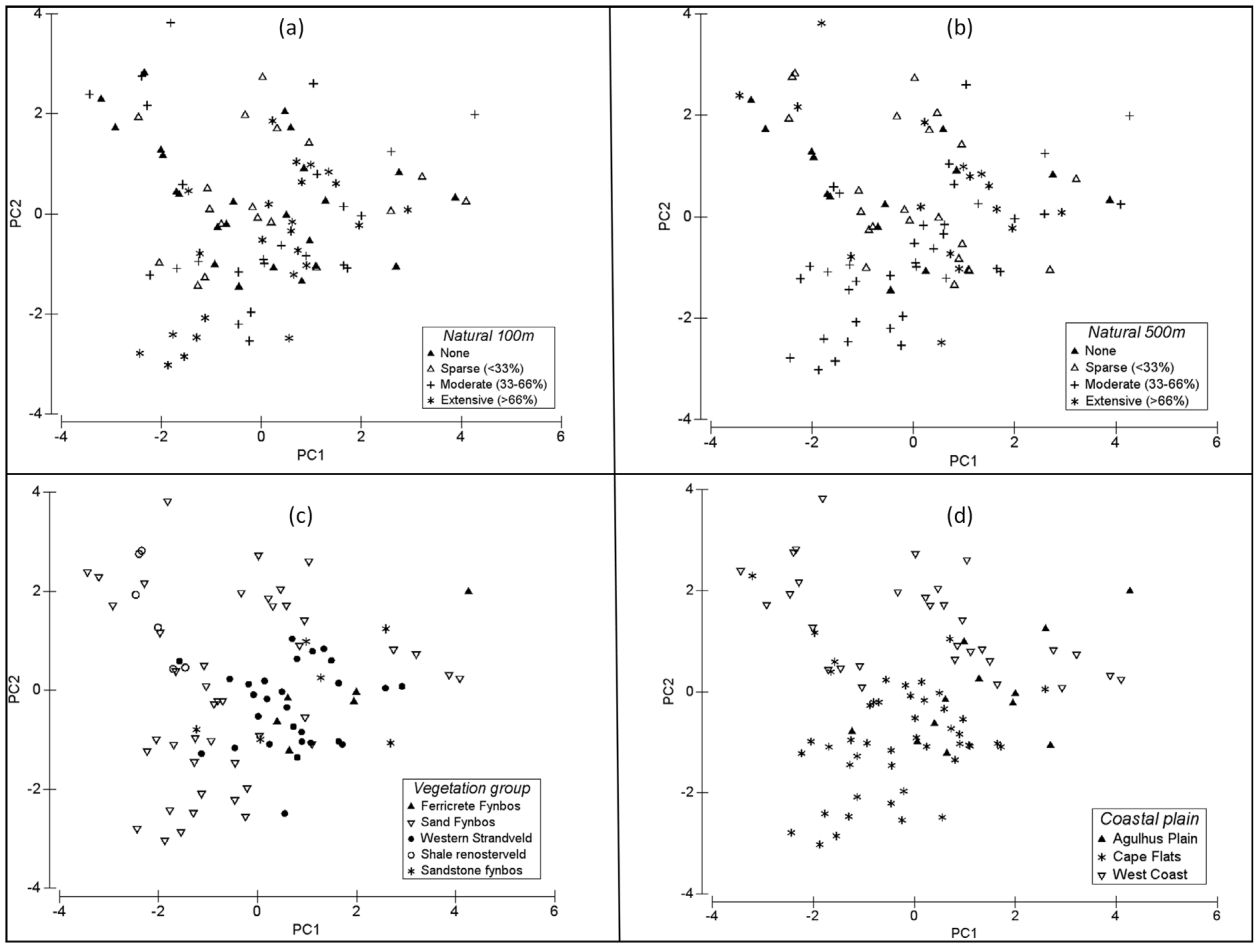
Principal Components Analysis ordinations of the physico-chemical variables (normalized) for all study sites (n = 90). The first two principal component axes are displayed. Sites are coded according to: (a) the areal cover of natural (indigenous) vegetation within a 100 m radius of each wetland edge; (b) the areal cover of natural (indigenous) vegetation within a 500 m radius of each wetland edge; (c) the natural vegetation type either presently or historically surrounding each wetland; and (d) the three broad coastal plains covered in this study.

Although the variables ‘Natural 100 m’ and ‘Natural 500 m’ were significantly related to physico-chemical conditions (Table 2), the patterns are not obvious in the PCA plots (Figure 2a–b) with no clear grouping according to the different levels of natural vegetation within 100 and 500 m. The PCA does not allow visualization of patterns with the effects of covariables partialled out (e.g. time, latitude), and thus may limit its usefulness in visualizing the effects of habitat transformation when covariables are involved in the analysis. In terms of physico-chemical constituents, sites showed better grouping according to the vegetation types in which they would naturally occur (i.e. the wetland clusters), but none of the vegetation types formed clusters that were clearly separated from the rest of the groups (Figure 2c). Variation within certain groups was large, particularly for the Sand fynbos cluster, which covered the broadest area of sampling and showed by far the most variation in physico-chemical conditions, as evidenced by scatter in the PCA plot among sites for this cluster (Figure 2c). The Western strandveld was the second largest cluster, showing the second-highest levels of physico-chemical variation. With the exception of Sandstone fynbos, which showed considerable variation among few sites, the remaining small wetland clusters displayed correspondingly lower levels of variation than for the bigger clusters. Physico-chemical conditions among the three broad coastal plain areas appear to be more distinguishable, although there is some overlap among areas towards the centre of the ordination (Figure 2d).

### Univariate patterns

pH, phosphates, dissolved oxygen and turbidity were negatively related to indigenous vegetation cover within both 100 and 500 m radii of wetlands (Table 3). With the exception of turbidity, the same variables were positively related to invasive vegetation cover within 100 m of wetlands. Only dissolved oxygen concentrations were significantly related (positive slope) to invasive vegetation cover within 500 m. Phosphate concentrations in wetlands were positively related to agricultural cover within 100 m, whilst ammonium concentrations were negatively related to agricultural cover within 500 m. pH was positively related to urban cover within 100 m of wetlands, but none of the variables were significantly related to urban cover within 500 m. The relationships presented in Table 3, although significant, are generally weak, as inferred from the low amounts of explained variation in the response variables due to the habitat transformation predictor variables (partial r^2^ values mostly <0.20 i.e. 20%, and none >0.30). As observed for the multivariate regressions (Table 2), the percentages of explained variation due to the spatio-temporal covariables (see ‘r^2^ - Covariables’) in Table 3 were for the most part considerably higher than that explained by the habitat transformation predictor variables (see ‘Partial r^2^ – predictor’). Six of the univariate relationships were significant at α<0.01 (Table 3a, 3e, 3i, 3l, 3o, 3p) and only two relationships (Table 3a, 3i) were significant after sequential Bonferroni correction.

**Table 3:** Multiple linear regression models (a - p) of environmental response variables regressed against the habitat transformation variables (predictors), given the spatio-temporal covariables.

	Predictor variables	Response variables	β	SE	Partial r^2^ (Predictor)	t	Res. df	P	Covariables	r^2^ (Covariables)
a)	Natural 100 m	pH	−0.354	0.081	0.187	−4.374	83	<0.001***	Longitude, latitude, time, altitude, Western strandveld	0.329
b)	Natural 100 m	Phosphates	−0.214	0.083	0.073	−2.591	86	0.011	Longitude, latitude, Shale renosterveld	0.371
c)	Natural 100 m	Dissolved oxygen	−0.216	0.092	0.061	−2.357	86	0.021	Altitude, Ferricrete fynbos	0.256
d)	Natural 100 m	Turbidity	−0.163	0.070	0.060	−2.326	85	0.022	Longitude, latitude, Shale renosterveld	0.543
e)	Natural 500 m	pH	−0.261	0.087	0.097	−2.993	83	0.004**	Longitude, latitude, time, altitude, Western strandveld	0.365
f)	Natural 500 m	Dissolved oxygen	−0.229	0.093	0.066	−2.467	86	0.016	Altitude, Ferricrete fynbos	0.255
g)	Natural 500 m	Phosphates	−0.195	0.084	0.059	−2.317	86	0.023	Longitude, latitude, Shale renosterveld	0.377
h)	Natural 500 m	Turbidity	−0.152	0.071	0.051	−2.138	85	0.035	Longitude, latitude, Shale renosterveld	0.548
i)	Invaded 100 m	pH	0.391	0.088	0.229	4.423	66	<0.001***	Time, altitude, Western strandveld	0.295
j)	Invaded 100 m	Phosphates	0.251	0.098	0.090	2.570	67	0.012	Longitude, latitude	0.274
k)	Invaded 100 m	Dissolved oxygen	0.248	0.102	0.079	2.418	68	0.018	Altitude	0.209
l)	Invaded 500 m	Dissolved oxygen	0.318	0.102	0.123	3.108	69	0.003**	Altitude	0.191
m)	Agriculture 100 m	Phosphates	0.371	0.161	0.210	2.307	20	0.032	Latitude, Sand fynbos	0.410
n)	Agriculture 500 m	Ammonium	−0.399	0.149	0.285	−2.676	18	0.015	Latitude	0.332
o)	Urban 100 m	Turbidity	0.457	0.135	0.253	3.395	34	0.002**	Latitude	0.158
p)	Urban 100 m	pH	0.341	0.131	0.167	2.610	34	0.013	Sand fynbos	0.252

Only significant relationships are presented here (α = 0.05, with the exception of agriculture, where α = 0.10). To maximise parsimony, covariable subsets were pre-selected for each model using step-wise regression of each response variable on the full list of possible covariables (see Table 1). For each predictor variable, results are listed in decreasing order of relationship strength based on P values. Only partial relationships between the response and predictor variables are reported here, not the full model results. Significant relationships at α<0.01 (**) and after sequential Bonferroni correction (***) are also indicated.

Natural - indigenous vegetation; Invaded - alien invasive vegetation; Agriculture - agricultural land; Urban - urban area. The areal cover of these variables is represented within 100 and 500 m radii of each wetland edge, measured on an ordinal scale. Time - Number of days since the first sampling event; β – standardized regression coefficient; SE – standard error of regression coefficient; Partial r^2^ – coefficient of partial determination for each respective predictor variable; Res. df – residual degrees of freedom; r^2^ (Covariables)  =  Full model r^2^ - Partial r^2^ (predictor).

**Figure 3:**
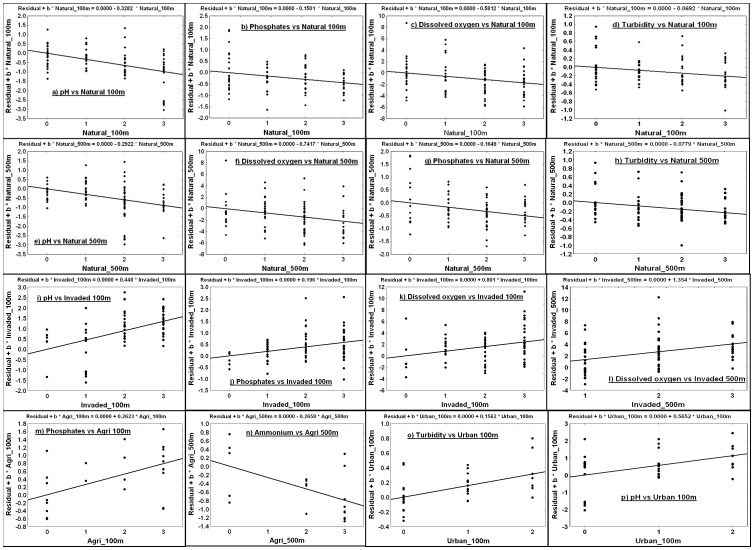
Partial residual plots displaying the relationships a – p presented in Table 3. Environmental response variables are depicted in relation to the habitat transformation variables (predictors, x axes), holding the spatio-temporal covariables constant. Natural - indigenous vegetation; Invaded - alien invasive vegetation; Agri - agricultural land; Urban – urban area. The areal cover of these variables is represented within 100 and 500 m radii of each wetland edge, measured on an ordinal scale: 0 – none; 1 – sparse; 2 – moderate; 3 – extensive. For more detailed information regarding each model, refer to Table 3. Note: The residuals on the vertical axis of each plot come from the regression of the response variable against all the predictors except the one of interest. The residuals for the horizontal axis of each plot come from the regression of the predictor variable of interest against all other predictors. Each residual scatterplot shows the relationship between a given univariate response variable and a predictor variable of interest, holding the other predictor variables constant. The regression equation for each relationship has been indicated, with each slope being equal to the non-standardized regression coefficient (b) in the full multiple regression model in which the parameter was included. ‘0.0000’ indicates that the intercept value is <0.0001.

The partial residual plots of Figure 3 offer visual representation of the regression relationships reported in Table 3, holding the covariables constant. Apparent in most of the plots is the considerable amount of vertical (Y axis) scatter in the residual points, which accounts for the low partial r^2^ values observed in Table 3 and shows that relationships were generally weak. The plots also allow identification of outliers or groups of high leverage points. The pattern for pH appears to be highly leveraged by five very low pH sites occurring in one particular area on the Cape Flats, visible at the bottom of the plots in Figure 3a, 3e, 3i and 3p. To test their influence, a *post hoc* analysis was run without these sites and revealed that the partial relationships between pH and natural vegetation cover within 100 and 500 m remained significant at α = 0.05, but were substantially weaker (‘Natural 100 m’: t_80_ = −2.124, P = 0.037, partial r^2^ = 0.053; ‘Natural 500 m’: t_80_ = −1.994, P = 0.049, partial r^2^ = 0.047). Partial relationships between pH and invasive vegetation cover within 100 m, and between pH and urban cover within 100 m, were rendered non-significant by exclusion of these sites from the models (‘Invaded 100 m’: t_61_ = 1.303, P = 0.198, partial r^2^ = 0.027; ‘Urban 100 m’: t_29_ = −0.187, P = 0.853, partial r^2^ = 0.001), indicating a strong influence of these sites on the regressions.

Relationships between phosphate concentrations and habitat transformation (Figure 3b, 3g, 3j and 3 m) showed high phosphate values associated with several of the extensively transformed wetlands, which may have influenced the reliability of these trends. Examination of Cook's distances for these models did not indicate that any of these high phosphate values had undue leverage on the trends (the maximum Cook's D_i_ value was 0.228). Dissolved oxygen concentrations showed similar patterns as for phosphates, although only one outlier was clearly apparent in these plots (Figure 3c and 3f, Figure 3k and 3l). Cook's distances once again indicated that no points had particularly undue leverage in these models (maximum Cook's D_i_ value: 0.373). Turbidity displayed weak linear trends with the ‘Natural 100 m’ and ‘Natural 500 m’ variables (Figure 3d and 3h respectively), with low gradient slopes and considerable spread in the residual points on either side of the regression line. The positive relationship between turbidity levels and urban cover within 100 m (Figure 3o) was clearer and showed less scatter among points.

## Discussion

### Indigenous vegetation cover

The multivariate regression relationships presented in this study indicate that human transformation of the landscape surrounding temporary depression wetlands in the south-western Cape is associated with physico-chemical conditions in these wetlands. This statement refers to overall transformation of adjacent habitats as represented by the remaining indigenous vegetation cover. The pattern appears to be slightly stronger for habitat transformation taking place within 100 m of wetlands than for 500 m, although significant trends were seen at both scales. The contribution to the percentage variation in physico-chemical conditions explained by variables representing the different types of habitat transformation was very low (2.08–5.57%, Table 2), despite being significant in some cases. This explained variation was generally in the region of one order of magnitude lower than that explained by the spatio-temporal covariables in the multivariate models (35.47–63.71%, Table 2). At the broad scale of this study, the primary influence on physico-chemical conditions in wetlands thus appeared to come from spatio-temporal factors, although a significant signal was still detected for certain habitat transformation factors over and above the spatio-temporal influence. This indicates firstly, that spatio-temporal variation in physico-chemical conditions is high for these wetlands, and secondly, that habitat transformation has played a meaningful role (albeit relatively weak in comparison to that of spatio-temporal factors) in altering the physico-chemistry of these wetlands, as was hypothesized at the outset of this study.

Our results are in line with those of a similar study by Declerck et al. [20] on the water quality of 99 small permanent ponds (natural and artificial) affected by agriculture in Belgium. These authors recorded land use at multiple spatial scales up to 3.2 km around ponds and found that the maximum amount of variation in a set of physico-chemical variables explained by crop land was 2.3% and by the amount of indigenous forest cover was 4%, both measured at a scale of 100 m around ponds and both were statistically significant. Their study also corroborates our finding that habitat transformation influences on the physico-chemistry of small, isolated wetlands appear to be strongest within 100 m of wetlands, although only a slight difference was found between the 100 and 500 m scales in this study. Given that small isolated wetlands have been shown elsewhere to drain localised catchments, they would not be expected to be affected by broader catchment-scale processes, as might be the case for rivers or lakes [56]. This may well explain why the strongest relationships between wetland physico-chemical conditions and surrounding land cover are reported at the 100 m scale in this study and that of Declerck et al. [20].

Negative linear relationships were reported between surrounding indigenous vegetation cover within 100 and 500 m of wetlands and pH, phosphates, dissolved oxygen and turbidity levels in these wetlands (Table 3). Turbidity has been shown to increase with transformation of the surrounding landscape for other wetland ecosystems, particularly as a result of sedimentation from agricultural or urban runoff [16], [20]. Replacement of natural vegetation often leads to de-stabilization of soils [21] and thus various forms of habitat transformation could be responsible for increased sediment input to wetlands through increased surface water flows during rain events. The negative relationship between wetland pH and natural vegetation cover within 100 m is most likely an effect of removing fynbos, which is known to release acidic leachates into the soil [34], [57]–[59]. The resultant physico-chemical effect would be an increase in the pH of wetlands as surrounding fynbos is lost. Bird et al. [34] found that the replacement of indigenous Sand fynbos with alien vegetation caused a reduction of humic input to wetlands, which in turn affected other physico-chemical constituents such as pH. They hypothesized that the alteration of pH with transformation of the landscape is only likely to occur in areas where soils are naturally acidic and the vegetation type is sclerophyllous fynbos. With reference to the current study, the Western strandveld cluster occurs on naturally alkaline soils due to the intrusion of calcareous sediments of marine origin [40], and the vegetation is not sclerophyllous, but dominated by succulents. Replacing this vegetation type with alien vegetation would not lead to effects on soil or surface water pH. However, Sand fynbos is a vegetation type that occurs on well-leached, naturally acidic soils and the vegetation itself is sclerophyllous, containing high levels of acidic tannins as a defence against herbivory [40]. Therefore, the loss of fynbos in this area can be hypothesized to raise soil and surface water pH. Closer inspection of Figure 3a and 3e reveals that five of the sites appeared to have a high leverage on the strength of the trend between indigenous vegetation cover and the pH of wetlands. These sites occurred inside the Kenilworth racetrack on the Cape Flats and were among the most pristine wetlands in the Sand fynbos cluster, due to an historical lack of disturbance inside the racetrack [60]–[64]. The low pH values are thus most likely a real reflection of the vast amount of undisturbed fynbos vegetation surrounding these sites. Evidence presented in this study provides further confirmation of the trend reported by Bird et al. [34], although the effect appears to be patchy at the broader scale and driven by sites occurring within Sand fynbos. One cannot expect to observe this relationship in areas where the natural vegetation type does not contain acidic tannins.

The negative relationships between surrounding indigenous vegetation cover and dissolved oxygen levels were surprising given that previous studies have found human disturbance of the landscape to be generally associated with decreased levels of oxygen and increased levels of nutrients in aquatic ecosystems (e.g. [65]–[67]). Further investigation is required to establish any underlying causes in this regard. The negative association between phosphate concentrations and natural vegetation cover within 100 and 500 m could be due to the effects of alien vegetation, agriculture or urban development.

### Alien invasive vegetation

Considering the three types of habitat transformation separately, only invasive vegetation cover was significantly related to physico-chemical conditions at both 100 and 500 m spatial scales and thus appears to be an influential form of habitat transformation. Using various modelling approaches, Rouget et al. [28] predicted that between 27.2 and 30% of remaining untransformed habitat in the Cape Floristic Region is likely to be invaded by alien plants over the next 20 years (i.e. from the time of their study). Our results suggest that this predicted spread of alien invasive plants into untransformed areas in the near future could impact significantly on temporary wetland environments occurring in those areas without ‘polluting’ or physically altering them. Our results corroborate those of Bird et al. [34] in suggesting that terrestrial alien plants are indeed affecting the water quality of aquatic ecosystems in the region, despite numerous research efforts that have focussed only on water quantity effects of alien plants. Furthermore this has importance in the light of changes in biotic assemblages that could potentially be induced by these physico-chemical effects, given the potential importance of physico-chemistry in structuring biotic assemblages such as invertebrates.

The positive association between dissolved oxygen concentrations in wetlands and surrounding invasive vegetation cover within 100 and 500 m was difficult to explain and no literature appears to report similar findings. Further investigation is required to explore possible mechanisms governing this trend, although it should be noted that these relationships were not convincing as reflected by the low partial r^2^ values (0.079 and 0.123 at 100 and 500 m respectively, Table 3). Phosphate concentrations (Appendix S3, S4 in File S1) were positively related to invasive vegetation cover within 100 m, but not 500 m, suggesting a localised nutrient input from invasive vegetation into groundwater. Once again the relationship was weak as judged by the small extent of explained variation in phosphates due to the predictor variable ‘Invaded 100 m’ (partial r^2^ = 0.090). Bird et al. [34] also reported a significant positive relationship between alien vegetation cover within 100 m of wetlands and water column phosphate concentrations in temporary wetlands. A possible mechanism governing this trend is the elevation of soil phosphorus in adjacent terrestrial soils due to infestation by alien shrubs, which may then leach into wetlands. This is postulated based on the findings of Witkowski and Mitchell [68], who reported a significant increase in soil phosphorus in stands of *Acacia saligna* (also the dominant invader in the current study) compared to surrounding natural lowland fynbos vegetation. It was established that this was due to higher litterfall from acacias, which released leaves into the soil with significantly higher phosphorus content than those of lowland fynbos vegetation [68].

The relatively strong (P = 0.001, partial r^2^ = 0.229, Table 3) positive relationship between pH and alien vegetation cover within 100 m is most likely a consequence of the loss of natural vegetation, which accompanies the transformation of habitats by invasive alien vegetation. As discussed earlier, we hypothesize that the loss of natural vegetation in the Sand fynbos area would cause an increase in soil and surface water pH through the loss of acidic tannins that characterise natural fynbos ecosystems. The predominant disturbance type in this area was alien vegetation and thus it was positively associated with levels of pH, even though it is not expected that alien vegetation itself raises the soil pH, but rather that it is associated with higher levels of pH as a consequence of the loss of naturally acidic vegetation to the system.

### Agriculture

Previous studies, mostly on permanent wetlands, have indicated that agriculture has significant impacts on the water chemistry of wetlands (e.g. [20], [21], [25], [69]), whilst no significant effects were detected in this study. This may, to some extent, be an artefact of the relatively small sample size for the agricultural datasets (100 m scale: n = 24; 500 m scale: n = 21), which reduces the statistical power to detect an effect [70]. The primary agricultural areas of the study region occur mostly on relatively fertile shale soils [40], where wheat agriculture has transformed the landscape so intensively that least impaired wetlands were difficult to find and it was necessary to search for small fragments of remaining natural vegetation that also happened to house temporary wetlands. In the Sand fynbos cluster, least impaired sites were not too difficult to find, and these were compared with sites occurring within pasture areas (the predominant form of agriculture in this area). However, the difficulty in this case was in finding enough sites within moderately and extensively transformed pasture areas. Our data on agriculture is thus limited and although no effect on wetland physico-chemistry was found, this should be interpreted with caution until a larger set of data is available. The lack of un-impacted depression wetlands that could be found within the extensively transformed wheat farming areas highlights the plight of these wetlands in lowland agricultural areas.

### Urban development

The association between urban cover within 100 m and physico-chemical conditions in the studied wetlands is in line with the few previous studies which have addressed the topic for temporary wetlands [18], [24], however certain affected variables appear to be different. The significant positive relationship between amount of urban area (within 100 m in this study) and wetland turbidity has been reported elsewhere [24], [71] and could be attributed to sedimentation from increased surface runoff, amongst other factors. The positive relationship between pH and urban cover within 100 m is once again likely to be due to the rise in pH associated with the loss of fynbos vegetation, as habitat is converted to urban surfaces. It was surprising that nutrient concentrations were not associated with surrounding urban cover, as previous literature has reported this for other temporary wetland systems [18], [24] and it was expected that the major form of disturbance for urban-exposed wetlands would be in the form of increased nutrient levels. Furthermore, it was expected that effects of urban development would extend beyond 100 m, given the intensity of this land use, and that a significant association would have been found between physico-chemical conditions and urban cover within 500 m. Follow-up work focussing on urban-encroached temporary wetlands in the area may help to establish why nutrient levels are not elevated in urban-impacted wetlands relative to unimpacted wetlands.

### Spatial patterns of wetland physico-chemistry

The PCA ordinations indicated that the spatial scale of sampling was positively associated with the amount of variation in physico-chemical conditions in the temporary wetlands. Although wetland clusters did not clearly separate out on the basis of their physico-chemical constituents, the amount of variation within each cluster was linked to the spatial area covered. The ordinations further indicated that the spatial scale with the clearest pattern of influence on the physico-chemical variables was at the level of broad latitudinal regions (Agulhus Plain, Cape Flats and West coast, Figure 2d). This appears to be consistent with the above-mentioned pattern of increased variation with increased spatial scale and reinforces the pattern of a link between spatial extent of sampling and increasing variation of physico-chemical conditions in the region. This is perhaps not surprising given that one expects more variation in physico-chemistry as the area sampled broadens, due to an associated increased variation in natural environmental factors such as geology and local climate. However, very little information exists on these basic aspects of spatial variation of environmental conditions in temporary wetlands of the region (but see [15], [34], [72]) and thus it is important to document such patterns. There is a certain degree of confounding from temporal differences between clusters (they were sampled sequentially), which cannot be accounted for in the PCA ordinations, but which were partialled out of the multiple regression models.

### Conclusions and recommendations

Significant physico-chemical signals (both multivariate and univariate) for effects of habitat transformation on temporary wetlands were detected over and above strong spatio-temporal influences in this study, confirming the hypothesis stated at the outset. We acknowledge that the dataset upon which our conclusions have been drawn does contain a degree of spatial and temporal confounding due to the sampling of wetlands over a period of several months and over a fairly large geographical area. However, this was largely unavoidable given the scale of the study and we have best accounted for spatio-temporal confounding through explicit incorporation of covariables into statistical models. Further studies at smaller spatial scales and better accounting for temporal variation are suggested to elucidate more specific information on the nature of the impacts of habitat transformation on temporary wetland ecosystems (e.g. [34]). The potential knock-on effects on wetland biota of the region due to physico-chemical changes associated with habitat transformation should also be investigated to help further understand the extent of ecosystem impacts (e.g. macroinvertebrate assemblages, [39], [73]). Our data indicate that the physico-chemical environment of these temporary wetlands is significantly influenced by human transformation of natural habitat within adjacent landscapes (<500 m). Relationships were generally stronger at the scale of 100 m around wetlands than for 500 m, indicating that preservation of narrow buffer strips of indigenous vegetation around these wetlands may afford significant protection of water quality. Restoration of even small fragments of terrestrial vegetation surrounding temporary wetlands is likely to yield significant improvements in water quality towards the original least impaired state.

## Supporting Information

File S1 **This file contains Appendix S1–S4.** Appendix S1. Data for the candidate covariables incorporated into the analyses of this study. Time was incorporated as a quantitative covariable measured as days since first sampling event (‘date sampled’ is provided for general reference). dd - decimal degrees. Appendix S2. Ordinal scores for each type of land cover around wetlands used to proxy habitat transformation gradients. Natural – indigenous vegetation; Invaded – alien invasive vegetation; Agriculture – land converted to agriculture; Urban – land converted to urban surfaces. The areal cover of these categories was scored within 100 and 500 m radii of each wetland edge using an ordinal scale: 0 - none; 1 – sparse cover (<33%); 2 – moderate cover (33–66%); 3 – extensive cover (>66%). Appendix S3. Environmental variables measured in this study. CV – complex vegetation biotope; SV – simple vegetation biotope; OW – open water biotope; BU – benthic un-vegetated biotope; TSA – total surface area; Max. depth – maximum depth. Appendix S4. Summary statistics of the physico-chemical variables (untransformed data) collected in this study, reported per wetland cluster (defined by terrestrial vegetation group). EC – electrical conductivity.
(DOCX)
