# Supplementary material for: Wetlands in Changed Landscapes: The Influence of Habitat Transformation on the Physico-Chemistry of Temporary Depression Wetlands
Source: PLoS One. 2014 Feb 12;9(2):e88935. doi: 10.1371/journal.pone.0088935 (PMC3923058; doi:10.1371/journal.pone.0088935)
Supplement: File S1 — This file contains Appendix S1–S4. Appendix S1. Data for the candidate covariables incorporated into the analyses of this study. Time was incorporated as a quantitative covariable measured as days since first sampling event (‘date sampled’ is provided for general reference). dd - decimal degrees. Appendix S2. Ordinal scores for each type of land cover around wetlands used to proxy habitat transformation gradients. Natural – indigenous vegetation; Invaded – alien invasive vegetation; Agriculture – land converted to agriculture; Urban – land converted to urban surfaces. The areal cover of these categories was scored within 100 and 500 m radii of each wetland edge using an ordinal scale: 0 - none; 1 – sparse cover (<33%); 2 – moderate cover (33–66%); 3 – extensive cover (>66%). Appendix S3. Environmental variables measured in this study. CV – complex vegetation biotope; SV – simple vegetation biotope; OW – open water biotope; BU – benthic un-vegetated biotope; TSA – total surface area; Max. depth – maximum depth. Appendix S4. Summary statistics of the physico-chemical variables (untransformed data) collected in this study, reported per wetland cluster (defined by terrestrial vegetation group). EC – electrical conductivity. (DOCX) [file pone.0088935.s001.docx]

**Appendix S1.** Data for the candidate covariables incorporated into the analyses of this study. Time was incorporated as a quantitative covariable measured as days since first sampling event (‘date sampled’ is provided for general reference). dd - decimal degrees.

| Site code | Date sampled | Latitude (dd) | Longitude (dd) | Time (days) | Altitude (m) | Vegetation group (wetland cluster) |
| --- | --- | --- | --- | --- | --- | --- |
|  |  |  |  |  |  |  |
| FER01 | 11/10/2007 | -34.6981 | 19.7202 | 80 | 100 | Ferricrete fynbos |
| FER02 | 11/10/2007 | -34.6970 | 19.7207 | 80 | 100 | Ferricrete fynbos |
| FER03 | 11/10/2007 | -34.6977 | 19.7276 | 80 | 92 | Ferricrete fynbos |
| FER04 | 11/10/2007 | -34.7214 | 19.7566 | 80 | 26 | Ferricrete fynbos |
| FER05 | 13/10/2007 | -34.5954 | 19.9589 | 82 | 30 | Ferricrete fynbos |
| FER06 | 14/10/2007 | -34.7108 | 19.9306 | 83 | 1 | Ferricrete fynbos |
| SAN01 | 23/07/2007 | -34.0379 | 18.7250 | 0 | 11 | Sand fynbos |
| SAN02 | 23/07/2007 | -34.0363 | 18.7253 | 0 | 11 | Sand fynbos |
| SAN03 | 08/09/2007 | -33.0083 | 18.3515 | 47 | 30 | Sand fynbos |
| SAN04 | 08/09/2007 | -33.0842 | 18.3971 | 47 | 50 | Sand fynbos |
| SAN05 | 20/09/2007 | -33.0870 | 18.3977 | 59 | 44 | Sand fynbos |
| SAN06 | 20/09/2007 | -33.0853 | 18.3981 | 59 | 50 | Sand fynbos |
| SAN07 | 20/09/2007 | -33.0724 | 18.3719 | 59 | 62 | Sand fynbos |
| SAN08 | 07/09/2007 | -33.5146 | 18.6546 | 46 | 120 | Sand fynbos |
| SAN09 | 07/09/2007 | -33.5449 | 18.6356 | 46 | 97 | Sand fynbos |
| SAN10 | 07/09/2007 | -33.5912 | 18.6059 | 46 | 90 | Sand fynbos |
| SAN11 | 11/08/2007 | -34.0308 | 18.7249 | 19 | 13 | Sand fynbos |
| SAN12 | 25/07/2007 | -33.9995 | 18.4854 | 2 | 25 | Sand fynbos |
| SAN13 | 25/07/2007 | -33.9984 | 18.4857 | 2 | 24 | Sand fynbos |
| SAN14 | 25/07/2007 | -33.9981 | 18.4873 | 2 | 23 | Sand fynbos |
| SAN15 | 25/07/2007 | -34.0000 | 18.4862 | 2 | 25 | Sand fynbos |
| SAN16 | 25/07/2007 | -34.0004 | 18.4836 | 2 | 27 | Sand fynbos |
| SAN17 | 31/07/2007 | -33.9988 | 18.4820 | 8 | 27 | Sand fynbos |
| SAN18 | 31/07/2007 | -33.9978 | 18.4822 | 8 | 29 | Sand fynbos |
| SAN19 | 31/07/2007 | -33.9971 | 18.4827 | 8 | 27 | Sand fynbos |
| SAN20 | 31/07/2007 | -33.9963 | 18.4821 | 8 | 29 | Sand fynbos |
| SAN21 | 31/07/2007 | -33.9959 | 18.4838 | 8 | 24 | Sand fynbos |
| SAN22 | 31/07/2007 | -33.9942 | 18.4836 | 8 | 27 | Sand fynbos |
| SAN23 | 31/07/2007 | -33.9945 | 18.4848 | 8 | 25 | Sand fynbos |
| SAN24 | 01/08/2007 | -33.9964 | 18.4848 | 9 | 26 | Sand fynbos |
| SAN25 | 01/08/2007 | -33.9926 | 18.4873 | 9 | 25 | Sand fynbos |
| SAN26 | 01/08/2007 | -33.9932 | 18.4838 | 9 | 26 | Sand fynbos |
| SAN27 | 01/08/2007 | -33.9932 | 18.4832 | 9 | 27 | Sand fynbos |
| SAN28 | 04/09/2007 | -34.0038 | 18.4875 | 43 | 26 | Sand fynbos |
| SAN29 | 08/08/2007 | -34.0540 | 18.5053 | 16 | 9 | Sand fynbos |
| SAN30 | 08/08/2007 | -34.0487 | 18.5104 | 16 | 13 | Sand fynbos |
| SAN31 | 08/08/2007 | -34.0381 | 18.5356 | 16 | 18 | Sand fynbos |
| SAN32 | 08/08/2007 | -34.0401 | 18.5340 | 16 | 18 | Sand fynbos |
| SAN33 | 10/08/2007 | -34.0274 | 18.5397 | 18 | 19 | Sand fynbos |
| SAN34 | 17/09/2007 | -33.7096 | 18.4544 | 56 | 6 | Sand fynbos |
| SAN35 | 17/09/2007 | -33.6902 | 18.4547 | 56 | 12 | Sand fynbos |
| SAN36 | 17/09/2007 | -33.7033 | 18.4687 | 56 | 16 | Sand fynbos |
| SAN37 | 17/09/2007 | -33.7001 | 18.4683 | 56 | 20 | Sand fynbos |
| SAN38 | 20/09/2007 | -32.8073 | 18.3598 | 59 | 37 | Sand fynbos |
| **(Continued overleaf)** | |  |  |  |  |  |
| SAN39  **Appendix S1. (continued)** | 21/09/2007 | -32.7682 | 18.2391 | 60 | 23 | Sand fynbos |
| SAN40 | 21/09/2007 | -32.7704 | 18.2308 | 60 | 15 | Sand fynbos |
| SAN41 | 22/09/2007 | -33.3411 | 18.1848 | 61 | 5 | Sand fynbos |
| SAN42 | 22/09/2007 | -33.3404 | 18.1837 | 61 | 4 | Sand fynbos |
| SAN43 | 22/09/2007 | -33.4038 | 18.2793 | 61 | 63 | Sand fynbos |
| SAN44 | 22/09/2007 | -33.4035 | 18.2796 | 62 | 63 | Sand fynbos |
| SST01 | 10/10/2007 | -34.7405 | 19.6794 | 79 | 3 | Sandstone fynbos |
| SST02 | 10/10/2007 | -34.7407 | 19.6783 | 79 | 6 | Sandstone fynbos |
| SST03 | 10/10/2007 | -34.7387 | 19.6407 | 79 | 5 | Sandstone fynbos |
| SST04 | 10/10/2007 | -34.7397 | 19.7325 | 79 | 7 | Sandstone fynbos |
| SST05 | 10/10/2007 | -34.7257 | 19.7334 | 79 | 13 | Sandstone fynbos |
| SST06 | 12/10/2007 | -34.7526 | 19.8017 | 81 | 14 | Sandstone fynbos |
| SHA01 | 05/09/2007 | -32.7723 | 18.8183 | 44 | 126 | Shale renosterveld |
| SHA02 | 05/09/2007 | -32.7024 | 18.8361 | 44 | 125 | Shale renosterveld |
| SHA03 | 06/09/2007 | -32.6886 | 18.9327 | 45 | 159 | Shale renosterveld |
| SHA04 | 06/09/2007 | -32.6776 | 18.9345 | 45 | 151 | Shale renosterveld |
| SHA05 | 06/09/2007 | -32.6409 | 18.8909 | 45 | 138 | Shale renosterveld |
| SHA06 | 07/09/2007 | -32.9016 | 18.7989 | 46 | 120 | Shale renosterveld |
| WES01 | 16/08/2007 | -34.0414 | 18.7241 | 24 | 9 | Western strandveld |
| WES02 | 17/08/2007 | -34.0374 | 18.7226 | 25 | 12 | Western strandveld |
| WES03 | 11/08/2007 | -34.0346 | 18.7216 | 19 | 11 | Western strandveld |
| WES04 | 11/08/2007 | -34.0364 | 18.7214 | 19 | 12 | Western strandveld |
| WES05 | 04/10/2007 | -34.0119 | 18.6643 | 73 | 34 | Western strandveld |
| WES06 | 04/10/2007 | -34.0129 | 18.6644 | 73 | 32 | Western strandveld |
| WES07 | 04/10/2007 | -34.0119 | 18.6675 | 73 | 29 | Western strandveld |
| WES08 | 05/10/2007 | -33.9845 | 18.6606 | 74 | 38 | Western strandveld |
| WES09 | 05/10/2007 | -33.9889 | 18.6593 | 74 | 33 | Western strandveld |
| WES10 | 23/07/2007 | -34.0490 | 18.7170 | 0 | 14 | Western strandveld |
| WES11 | 23/07/2007 | -34.0455 | 18.7226 | 0 | 9 | Western strandveld |
| WES12 | 23/07/2007 | -34.0435 | 18.7248 | 0 | 10 | Western strandveld |
| WES13 | 10/09/2007 | -33.6872 | 18.4356 | 49 | 6 | Western strandveld |
| WES14 | 10/09/2007 | -33.6853 | 18.4349 | 49 | 5 | Western strandveld |
| WES15 | 10/09/2007 | -33.6842 | 18.4368 | 49 | 8 | Western strandveld |
| WES16 | 10/09/2007 | -33.6855 | 18.4374 | 49 | 10 | Western strandveld |
| WES17 | 10/09/2007 | -33.6869 | 18.4369 | 49 | 8 | Western strandveld |
| WES18 | 10/09/2007 | -33.6922 | 18.4386 | 49 | 7 | Western strandveld |
| WES19 | 08/08/2007 | -34.0581 | 18.5046 | 16 | 10 | Western strandveld |
| WES20 | 08/08/2007 | -34.0585 | 18.5035 | 16 | 7 | Western strandveld |
| WES21 | 08/08/2007 | -34.0581 | 18.5001 | 16 | 7 | Western strandveld |
| WES22 | 10/08/2007 | -34.0675 | 18.4950 | 18 | 6 | Western strandveld |
| WES23 | 10/08/2007 | -34.0711 | 18.4983 | 18 | 7 | Western strandveld |
| WES24 | 10/08/2007 | -34.0696 | 18.4980 | 18 | 8 | Western strandveld |
| WES25 | 23/07/2007 | -34.0044 | 18.6435 | 0 | 37 | Western strandveld |
| WES26 | 01/10/2007 | -34.0124 | 18.6813 | 70 | 28 | Western strandveld |
| WES27 | 01/10/2007 | -34.0090 | 18.6806 | 70 | 28 | Western strandveld |
| WES28 | 01/10/2007 | -34.0096 | 18.6785 | 70 | 18 | Western strandveld |
|  |  |  |  |  |  |  |

**Appendix S2.** Ordinal scores for each type of land cover around wetlands used to proxy habitat transformation gradients. Natural – indigenous vegetation; Invaded – alien invasive vegetation; Agriculture – land converted to agriculture; Urban – land converted to urban surfaces. The areal cover of these categories was scored within 100 and 500 m radii of each wetland edge using an ordinal scale: 0 - none; 1 – sparse cover (< 33%); 2 – moderate cover (33-66%); 3 – extensive cover (> 66%).

| Site code | Natural 100m | Invaded 100m | Agriculture 100m | Urban  100m | Natural 500m | Invaded 500m | Agricultural 500m | Urban  500m |
| --- | --- | --- | --- | --- | --- | --- | --- | --- |
| FER01 | 3 | 0 | 1 | 0 | 2 | 0 | 2 | 0 |
| FER02 | 3 | 0 | 1 | 0 | 2 | 0 | 2 | 0 |
| FER03 | 2 | 0 | 2 | 0 | 2 | 0 | 2 | 0 |
| FER04 | 3 | 0 | 0 | 0 | 3 | 1 | 0 | 0 |
| FER05 | 2 | 1 | 2 | 0 | 2 | 1 | 2 | 0 |
| FER06 | 2 | 0 | 2 | 0 | 2 | 1 | 2 | 0 |
| SAN01 | 1 | 3 | 0 | 0 | 1 | 3 | 0 | 1 |
| SAN02 | 0 | 2 | 0 | 2 | 1 | 2 | 0 | 2 |
| SAN03 | 2 | 2 | 0 | 0 | 3 | 1 | 0 | 0 |
| SAN04 | 3 | 1 | 0 | 0 | 3 | 1 | 0 | 0 |
| SAN05 | 2 | 2 | 0 | 0 | 3 | 1 | 0 | 0 |
| SAN06 | 2 | 2 | 0 | 0 | 3 | 1 | 0 | 0 |
| SAN07 | 1 | 0 | 3 | 0 | 1 | 1 | 3 | 0 |
| SAN08 | 0 | 3 | 1 | 0 | 0 | 3 | 1 | 0 |
| SAN09 | 1 | 1 | 3 | 0 | 1 | 1 | 3 | 0 |
| SAN10 | 1 | 3 | 0 | 0 | 1 | 3 | 0 | 0 |
| SAN11 | 0 | 3 | 0 | 0 | 0 | 3 | 0 | 0 |
| SAN12 | 2 | 2 | 0 | 1 | 2 | 2 | 0 | 1 |
| SAN13 | 1 | 3 | 0 | 1 | 2 | 2 | 0 | 1 |
| SAN14 | 3 | 1 | 0 | 0 | 2 | 2 | 0 | 1 |
| SAN15 | 1 | 3 | 0 | 0 | 2 | 2 | 0 | 1 |
| SAN16 | 2 | 2 | 0 | 0 | 2 | 2 | 0 | 1 |
| SAN17 | 2 | 2 | 0 | 0 | 2 | 2 | 0 | 1 |
| SAN18 | 3 | 1 | 0 | 0 | 2 | 2 | 0 | 1 |
| SAN19 | 3 | 1 | 0 | 0 | 2 | 2 | 0 | 1 |
| SAN20 | 2 | 2 | 0 | 0 | 2 | 2 | 0 | 1 |
| SAN21 | 3 | 0 | 0 | 0 | 2 | 2 | 0 | 1 |
| SAN22 | 2 | 2 | 0 | 0 | 2 | 2 | 0 | 1 |
| SAN23 | 3 | 1 | 0 | 0 | 2 | 2 | 0 | 1 |
| SAN24 | 3 | 1 | 0 | 0 | 2 | 2 | 0 | 1 |
| SAN25 | 0 | 3 | 0 | 1 | 1 | 2 | 0 | 2 |
| SAN26 | 2 | 2 | 0 | 0 | 2 | 2 | 0 | 1 |
| SAN27 | 2 | 2 | 0 | 0 | 2 | 2 | 0 | 1 |
| SAN28 | 1 | 3 | 0 | 0 | 1 | 3 | 0 | 1 |
| SAN29 | 0 | 2 | 0 | 2 | 1 | 1 | 0 | 3 |
| SAN30 | 0 | 3 | 0 | 1 | 0 | 1 | 0 | 3 |
| SAN31 | 0 | 2 | 1 | 2 | 0 | 2 | 1 | 2 |
| SAN32 | 0 | 3 | 1 | 1 | 0 | 2 | 1 | 2 |
| SAN33 | 0 | 0 | 3 | 0 | 0 | 1 | 3 | 1 |
| SAN34 | 1 | 3 | 0 | 0 | 1 | 3 | 0 | 0 |
| **(Continued overleaf)** | | | | | | | | |
| SAN35  **Appendix S2. (Continued)** | 0 | 3 | 0 | 0 | 0 | 2 | 0 | 2 |
| SAN36 | 0 | 3 | 0 | 1 | 0 | 2 | 0 | 2 |
| SAN37 | 0 | 2 | 0 | 2 | 0 | 2 | 0 | 2 |
| SAN38 | 1 | 2 | 2 | 0 | 1 | 2 | 2 | 0 |
| SAN39 | 0 | 1 | 3 | 0 | 0 | 1 | 3 | 0 |
| SAN40 | 0 | 1 | 3 | 0 | 1 | 1 | 3 | 0 |
| SAN41 | 1 | 3 | 0 | 0 | 2 | 2 | 0 | 0 |
| SAN42 | 2 | 2 | 0 | 0 | 2 | 2 | 0 | 0 |
| SAN43 | 1 | 3 | 0 | 0 | 1 | 3 | 0 | 1 |
| SAN44 | 1 | 3 | 0 | 0 | 1 | 3 | 0 | 1 |
| SST01 | 3 | 1 | 0 | 0 | 3 | 1 | 0 | 0 |
| SST02 | 2 | 2 | 0 | 0 | 2 | 2 | 0 | 0 |
| SST03 | 0 | 3 | 0 | 0 | 2 | 2 | 0 | 0 |
| SST04 | 3 | 1 | 0 | 0 | 3 | 1 | 0 | 0 |
| SST05 | 2 | 2 | 0 | 0 | 2 | 2 | 0 | 0 |
| SST06 | 0 | 3 | 0 | 0 | 1 | 3 | 0 | 0 |
| SHA01 | 3 | 1 | 0 | 0 | 2 | 1 | 2 | 0 |
| SHA02 | 1 | 0 | 3 | 0 | 1 | 0 | 3 | 0 |
| SHA03 | 0 | 0 | 3 | 0 | 0 | 0 | 3 | 0 |
| SHA04 | 2 | 0 | 2 | 0 | 1 | 0 | 3 | 0 |
| SHA05 | 0 | 0 | 3 | 0 | 1 | 0 | 3 | 0 |
| SHA06 | 0 | 0 | 3 | 0 | 0 | 0 | 3 | 0 |
| WES01 | 1 | 3 | 0 | 0 | 1 | 3 | 0 | 0 |
| WES02 | 0 | 3 | 0 | 1 | 1 | 3 | 0 | 1 |
| WES03 | 0 | 3 | 0 | 0 | 0 | 3 | 0 | 1 |
| WES04 | 0 | 3 | 0 | 1 | 0 | 3 | 0 | 1 |
| WES05 | 1 | 3 | 0 | 0 | 2 | 2 | 0 | 1 |
| WES06 | 1 | 3 | 0 | 0 | 2 | 2 | 0 | 1 |
| WES07 | 3 | 1 | 0 | 0 | 2 | 2 | 0 | 0 |
| WES08 | 3 | 0 | 0 | 0 | 2 | 2 | 0 | 0 |
| WES09 | 3 | 0 | 0 | 0 | 3 | 1 | 0 | 0 |
| WES10 | 2 | 2 | 0 | 1 | 1 | 2 | 0 | 2 |
| WES11 | 0 | 3 | 0 | 0 | 1 | 3 | 0 | 0 |
| WES12 | 1 | 3 | 0 | 0 | 1 | 3 | 0 | 0 |
| WES13 | 2 | 2 | 0 | 0 | 3 | 1 | 0 | 0 |
| WES14 | 2 | 2 | 0 | 0 | 3 | 1 | 0 | 0 |
| WES15 | 3 | 1 | 0 | 0 | 3 | 1 | 0 | 0 |
| WES16 | 3 | 0 | 0 | 0 | 3 | 1 | 0 | 0 |
| WES17 | 3 | 0 | 0 | 0 | 3 | 1 | 0 | 0 |
| WES18 | 3 | 0 | 0 | 1 | 2 | 1 | 0 | 2 |
| WES19 | 1 | 3 | 0 | 1 | 2 | 1 | 0 | 2 |
| WES20 | 2 | 2 | 0 | 1 | 2 | 1 | 0 | 2 |
| WES21 | 2 | 0 | 0 | 2 | 2 | 1 | 0 | 2 |
| WES22 | 3 | 1 | 0 | 0 | 3 | 1 | 0 | 1 |
| **(Continued overleaf)** | | | | | | | | |
| WES23  **Appendix S2. (Continued)** | 3 | 1 | 0 | 0 | 3 | 1 | 0 | 1 |
| WES24 | 3 | 1 | 0 | 0 | 3 | 1 | 0 | 1 |
| WES25 | 0 | 2 | 0 | 2 | 1 | 1 | 0 | 3 |
| WES26 | 3 | 1 | 0 | 1 | 2 | 1 | 0 | 2 |
| WES27 | 2 | 1 | 0 | 2 | 2 | 1 | 0 | 2 |
| WES28 | 2 | 1 | 0 | 2 | 2 | 1 | 0 | 2 |
|  |  |  |  |  |  |  |  |  |

**Appendix S3.** Environmental variables measured in this study. CV – complex vegetation biotope; SV – simple vegetation biotope; OW – open water biotope; BU – benthic un-vegetated biotope; TSA – total surface area; Max. depth – maximum depth

| Site code | pH | Conductivity (µS.cm^-1^) | Ave Temp. (°C) | Turbidity (NTU) | Oxygen (mg.L^-1^) | NO_3_^-^+NO_2_^-^ (μg.L^-1^) | PO_4_^3+^ (μg.L^-1^) | NH_4_^+^ (μg.L^-1^) | %CV | %SV | %OW | %BU | TSA  (m^2^) | Max. depth (cm) |
| --- | --- | --- | --- | --- | --- | --- | --- | --- | --- | --- | --- | --- | --- | --- |
| FER01 | 8.10 | 826 | 18.75 | 9.5 | 9.60 | 1.86 | 11.20 | 23.40 | 0 | 30 | 0 | 70 | 1963 | 22 |
| FER02 | 7.22 | 825 | 18.77 | 3.0 | 7.87 | 0.00 | 3.39 | 16.41 | 5 | 60 | 0 | 35 | 589 | 42 |
| FER03 | 7.05 | 1832 | 21.83 | 2.0 | 6.77 | 6.73 | 3.69 | 21.66 | 60 | 40 | 0 | 0 | 48381 | 70 |
| FER04 | 8.80 | 1786 | 22.42 | 4.0 | 10.33 | 1.99 | 4.11 | 8.57 | 60 | 20 | 20 | 0 | 14019 | 80 |
| FER05 | 10.12 | 17880 | 28.58 | 3.5 | 12.53 | 0.00 | 18.80 | 19.97 | 90 | 10 | 0 | 0 | 7540 | 15 |
| FER06 | 8.52 | 3143 | 20.97 | 12.0 | 10.10 | 0.00 | 1.58 | 17.21 | 70 | 0 | 0 | 30 | 29452 | 28 |
| SAN01 | 7.86 | 429 | 15.07 | 18.6 | 5.07 | 2.57 | 13.67 | 33.44 | 70 | 20 | 10 | 0 | 1257 | 45 |
| SAN02 | 8.57 | 258 | 19.40 | 2.1 | 10.03 | 0.88 | 9.38 | 26.00 | 100 | 0 | 0 | 0 | 471 | 25 |
| SAN03 | 7.04 | 570 | 17.32 | 4.4 | 1.20 | 28.95 | 2827.36 | 2314.41 | 10 | 5 | 85 | 0 | 1665 | 68 |
| SAN04 | 8.21 | 3697 | 18.73 | 14.7 | 8.50 | 0.87 | 510.89 | 105.21 | 15 | 15 | 70 | 0 | 19242 | 50 |
| SAN05 | 7.27 | 2733 | 17.57 | 4.0 | 1.38 | 10.55 | 615.00 | 1524.48 | 0 | 60 | 40 | 0 | 1885 | 25 |
| SAN06 | 7.57 | 3313 | 24.25 | 18.5 | 3.13 | 17.95 | 816.31 | 4231.53 | 40 | 10 | 50 | 0 | 1414 | 29 |
| SAN07 | 8.08 | 583 | 26.33 | 7.0 | 6.27 | 10.26 | 655.65 | 65.20 | 60 | 0 | 40 | 0 | 7461 | 39 |
| SAN08 | 6.90 | 306 | 18.20 | 7.4 | 1.73 | 11.62 | 213.88 | 2283.93 | 30 | 10 | 60 | 0 | 451 | 80 |
| SAN09 | 7.38 | 585 | 22.50 | 1.8 | 1.93 | 9.66 | 87.39 | 56.77 | 35 | 5 | 60 | 0 | 2827 | 44 |
| SAN10 | 7.25 | 261 | 20.45 | 4.5 | 4.60 | 11.24 | 37.11 | 47.04 | 60 | 0 | 40 | 0 | 1571 | 70 |
| SAN11 | 7.40 | 222 | 13.90 | 4.1 | 7.70 | 3.95 | 4.04 | 19.63 | 0 | 100 | 0 | 0 | 62832 | 45 |
| SAN12 | 6.68 | 239 | 11.88 | 5.3 | 3.70 | 44.19 | 8.57 | 33.23 | 70 | 0 | 30 | 0 | 611 | 28 |
| SAN13 | 6.78 | 231 | 11.41 | 3.8 | 3.15 | 5.30 | 45.79 | 39.47 | 20 | 70 | 10 | 0 | 298 | 50 |
| SAN14 | 6.55 | 169 | 12.33 | 1.4 | 5.00 | 2.12 | 5.42 | 19.46 | 90 | 0 | 10 | 0 | 5107 | 30 |
| SAN15 | 6.87 | 345 | 11.25 | 3.7 | 3.97 | 0.43 | 20.97 | 23.64 | 30 | 60 | 10 | 0 | 543 | 52 |
| SAN16 | 6.70 | 281 | 11.80 | 3.4 | 5.03 | 4.55 | 34.52 | 42.10 | 45 | 15 | 40 | 0 | 651 | 120 |
| SAN17 | 6.61 | 463 | 12.87 | 1.7 | 5.80 | 0.08 | 1.55 | 7.09 | 100 | 0 | 0 | 0 | 436 | 18 |
| SAN18 | 4.33 | 229 | 13.63 | 1.1 | 4.63 | 6.82 | 1.55 | 12.72 | 100 | 0 | 0 | 0 | 214 | 30 |
| SAN19 | 4.07 | 141 | 15.12 | 0.9 | 6.83 | 3.28 | 6.57 | 11.64 | 100 | 0 | 0 | 0 | 738 | 27 |
| SAN20 | 6.82 | 450 | 15.25 | 1.6 | 6.87 | 2.17 | 3.10 | 10.23 | 60 | 30 | 10 | 0 | 589 | 50 |
| SAN21 | 4.37 | 234 | 16.45 | 2.0 | 5.73 | 13.07 | 1.66 | 23.04 | 60 | 30 | 10 | 0 | 1542 | 87 |
| SAN22 | 6.44 | 184 | 15.10 | 1.0 | 6.17 | 0.67 | 2.90 | 15.26 | 35 | 45 | 20 | 0 | 5631 | 100 |
| SAN23 | 4.56 | 216 | 16.35 | 1.0 | 8.30 | 10.18 | 2.96 | 21.27 | 80 | 10 | 10 | 0 | 426 | 39 |
| SAN24 | 4.48 | 192 | 12.08 | 1.4 | 5.20 | 37.48 | 1.50 | 23.57 | 80 | 10 | 10 | 0 | 233 | 62 |
| SAN25 | 6.74 | 579 | 16.15 | 2.7 | 5.83 | 6.10 | 4.51 | 62.37 | 95 | 0 | 5 | 0 | 486 | 49 |
| SAN26 | 7.45 | 764 | 17.38 | 1.0 | 6.77 | 1.01 | 11.58 | 34.55 | 100 | 0 | 0 | 0 | 800 | 30 |
| SAN27 | 6.98 | 294 | 15.45 | 0.7 | 2.53 | 1.47 | 86.17 | 22.24 | 100 | 0 | 0 | 0 | 1081 | 40 |
| SAN28 | 8.31 | 114 | 19.20 | 1.5 | 11.07 | 1.33 | 7.14 | 11.70 | 50 | 0 | 20 | 30 | 721 | 40 |
| SAN29 | 7.66 | 958 | 15.52 | 2.8 | 4.00 | 5.87 | 121.89 | 14.58 | 30 | 20 | 50 | 0 | 8652 | 48 |
| SAN30 | 7.72 | 911 | 16.18 | 2.0 | 3.97 | 2.08 | 73.89 | 33.48 | 40 | 50 | 10 | 0 | 8234 | 120 |
| SAN31 | 7.79 | 1097 | 14.72 | 3.4 | 9.30 | 8241.59 | 1276.73 | 1087.33 | 50 | 10 | 40 | 0 | 18153 | 70 |
| SAN32 | 7.95 | 1048 | 15.88 | 2.6 | 7.47 | 801.02 | 1407.49 | 40.53 | 20 | 30 | 50 | 0 | 18153 | 150 |
| SAN33 | 8.00 | 986 | 13.68 | 2.0 | 8.60 | 602.28 | 440.30 | 24.68 | 20 | 10 | 70 | 0 | 2151 | 83 |
| SAN34 | 9.16 | 15617 | 18.70 | 2.3 | 14.33 | 0.01 | 119.92 | 6.70 | 95 | 0 | 0 | 5 | 716 | 38 |
| SAN35 | 8.41 | 12467 | 22.83 | 15.7 | 11.23 | 0.06 | 2.67 | 24.14 | 10 | 30 | 0 | 60 | 1395 | 27 |
| **(Continued overleaf)** | | | | | | | | | | | | | | |
| SAN36  **Appendix S3. (continued)** | 8.22 | 20833 | 22.18 | 2.5 | 16.70 | 0.02 | 14.31 | 8.22 | 0 | 60 | 40 | 0 | 1374 | 150 |
| SAN37 | 7.25 | 12983 | 23.13 | 17.5 | 7.13 | 1.36 | 23.90 | 28.38 | 20 | 60 | 0 | 20 | 707 | 21 |
| SAN38 | 8.27 | 7653 | 22.30 | 10.5 | 2.80 | 5.91 | 140.79 | 303.03 | 100 | 0 | 0 | 0 | 1414 | 30 |
| SAN39 | 8.71 | 2697 | 18.47 | 4.0 | 5.50 | 0.79 | 444.40 | 66.16 | 10 | 40 | 50 | 0 | 587 | 78 |
| SAN40 | 8.68 | 10773 | 21.65 | 9.0 | 9.10 | 4.39 | 594.89 | 73.38 | 0 | 0 | 0 | 100 | 3181 | 20 |
| SAN41 | 9.69 | 9190 | 22.12 | 4.0 | 15.47 | 0.12 | 1.67 | 8.21 | 60 | 0 | 40 | 0 | 518 | 150 |
| SAN42 | 8.42 | 8100 | 23.25 | 34.0 | 6.50 | 0.18 | 256.12 | 73.27 | 70 | 0 | 30 | 0 | 1257 | 30 |
| SAN43 | 7.93 | 7847 | 21.50 | 5.0 | 4.97 | 4.39 | 175.36 | 76.22 | 70 | 10 | 20 | 0 | 1963 | 34 |
| SAN44 | 8.27 | 876 | 20.93 | 6.7 | 6.77 | 1.71 | 68.31 | 58.76 | 80 | 10 | 10 | 0 | 1071 | 28 |
| SST01 | 6.71 | 883 | 14.97 | 2.5 | 6.53 | 18.50 | 9.62 | 73.75 | 70 | 30 | 0 | 0 | 1649 | 63 |
| SST02 | 7.74 | 2560 | 15.95 | 1.5 | 10.23 | 18.54 | 2.94 | 41.63 | 55 | 40 | 5 | 0 | 393 | 70 |
| SST03 | 7.78 | 2787 | 24.87 | 2.5 | 9.37 | 5.53 | 6.34 | 35.64 | 100 | 0 | 0 | 0 | 14137 | 14 |
| SST04 | 8.33 | 13460 | 23.67 | 12.5 | 8.80 | 8.91 | 9.42 | 40.99 | 100 | 0 | 0 | 0 | 17593 | 17 |
| SST05 | 9.74 | 3093 | 20.47 | 5.0 | 8.63 | 0.00 | 11.16 | 38.35 | 0 | 0 | 0 | 100 | 1571 | 10 |
| SST06 | 8.06 | 488 | 24.72 | 1.0 | 8.90 | 0.00 | 6.36 | 0.23 | 30 | 30 | 40 | 0 | 3004 | 56 |
| SHA01 | 7.27 | 353 | 17.03 | 9.8 | 5.87 | 14.58 | 66.27 | 98.39 | 40 | 40 | 20 | 0 | 15708 | 90 |
| SHA02 | 6.95 | 354 | 17.72 | 157.5 | 3.33 | 2.56 | 15.86 | 2803.87 | 0 | 60 | 40 | 0 | 11781 | 40 |
| SHA03 | 7.21 | 436 | 16.94 | 53.9 | 3.93 | 5.63 | 137.35 | 164.10 | 30 | 0 | 70 | 0 | 2513 | 50 |
| SHA04 | 7.35 | 260 | 19.72 | 38.1 | 4.17 | 5.92 | 999.41 | 1534.37 | 40 | 10 | 0 | 50 | 3711 | 38 |
| SHA05 | 7.72 | 265 | 18.25 | 713.0 | 6.57 | 47.65 | 579.56 | 130.81 | 40 | 10 | 50 | 0 | 1571 | 40 |
| SHA06 | 7.31 | 2730 | 12.31 | 439.5 | 3.83 | 0.06 | 12.03 | 61.72 | 0 | 70 | 30 | 0 | 3142 | 40 |
| WES01 | 7.94 | 3110 | 17.48 | 1.1 | 8.27 | 57.00 | 26.06 | 50.33 | 100 | 0 | 0 | 0 | 1257 | 35 |
| WES02 | 7.81 | 840 | 15.67 | 2.1 | 8.30 | 0.12 | 3.96 | 15.85 | 45 | 40 | 15 | 0 | 1885 | 45 |
| WES03 | 7.60 | 1158 | 14.80 | 44.2 | 7.07 | 0.71 | 4.46 | 2.24 | 30 | 20 | 50 | 0 | 942 | 80 |
| WES04 | 7.93 | 1740 | 15.52 | 1.8 | 6.40 | 2.39 | 166.28 | 68.35 | 40 | 40 | 20 | 0 | 589 | 80 |
| WES05 | 8.11 | 5093 | 18.40 | 2.0 | 5.53 | 3.16 | 14.10 | 34.33 | 5 | 95 | 0 | 0 | 3927 | 50 |
| WES06 | 9.01 | 3553 | 22.52 | 1.5 | 10.43 | 0.56 | 2.81 | 20.06 | 15 | 45 | 40 | 0 | 471 | 150 |
| WES07 | 8.19 | 3803 | 23.02 | 5.5 | 5.93 | 2.33 | 13.71 | 96.93 | 5 | 95 | 0 | 0 | 7854 | 20 |
| WES08 | 8.34 | 2373 | 18.93 | 1.0 | 2.37 | 1.82 | 7.91 | 15.30 | 34 | 33 | 33 | 0 | 9425 | 75 |
| WES09 | 8.06 | 1100 | 21.10 | 5.5 | 2.00 | 2.28 | 6.97 | 27.18 | 30 | 60 | 10 | 0 | 1257 | 80 |
| WES10 | 8.17 | 863 | 18.12 | 1.4 | 9.63 | 3.27 | 7.76 | 14.96 | 60 | 30 | 10 | 0 | 3927 | 45 |
| WES11 | 8.37 | 1026 | 16.38 | 4.6 | 11.33 | 2.77 | 37.06 | 38.51 | 30 | 45 | 25 | 0 | 1731 | 45 |
| WES12 | 7.63 | 8193 | 15.90 | 6.1 | 8.57 | 0.94 | 76.59 | 31.35 | 20 | 40 | 40 | 0 | 3310 | 80 |
| WES13 | 8.29 | 6453 | 18.47 | 1.9 | 9.47 | 1.56 | 9.29 | 34.97 | 60 | 0 | 40 | 0 | 3793 | 55 |
| WES14 | 8.20 | 9253 | 19.50 | 2.7 | 7.60 | 0.37 | 43.87 | 64.42 | 60 | 0 | 40 | 0 | 1885 | 35 |
| WES15 | 8.23 | 10640 | 22.38 | 1.7 | 5.63 | 1.26 | 6.33 | 54.11 | 70 | 0 | 30 | 0 | 707 | 35 |
| WES16 | 8.18 | 7723 | 22.55 | 1.2 | 4.97 | 1.47 | 14.51 | 59.75 | 70 | 0 | 30 | 0 | 4712 | 35 |
| WES17 | 8.64 | 3473 | 22.57 | 0.9 | 13.33 | 0.99 | 2.25 | 34.44 | 50 | 10 | 40 | 0 | 2199 | 45 |
| WES18 | 8.12 | 1178 | 23.73 | 8.8 | 3.33 | 0.91 | 4.28 | 38.75 | 30 | 70 | 0 | 0 | 3010 | 45 |
| WES19 | 7.43 | 1362 | 11.22 | 1.5 | 4.90 | 13.74 | 5.46 | 40.80 | 5 | 95 | 0 | 0 | 13435 | 48 |
| WES20 | 7.63 | 1311 | 12.85 | 1.6 | 6.97 | 12.14 | 9.45 | 19.32 | 50 | 30 | 20 | 0 | 4519 | 150 |
| WES21 | 7.33 | 4023 | 16.00 | 12.0 | 5.97 | 60.67 | 34.39 | 109.57 | 30 | 30 | 40 | 0 | 8488 | 150 |
| WES22 | 8.12 | 20833 | 13.53 | 0.9 | 8.40 | 1.64 | 6.36 | 17.64 | 20 | 40 | 40 | 0 | 1374 | 180 |
| **(Continued overleaf)** | | | | | | | | | | | | | | |
| WES23  **Appendix S3. (continued)** | 7.62 | 890 | 13.42 | 0.4 | 8.13 | 4.47 | 2.72 | 1.47 | 25 | 45 | 30 | 0 | 3000 | 150 |
| WES24 | 8.31 | 948 | 14.17 | 1.0 | 8.60 | 1.72 | 15.59 | 21.14 | 10 | 30 | 60 | 0 | 2356 | 150 |
| WES25 | 8.02 | 1146 | 17.17 | 1.3 | 6.90 | 0.13 | 2.10 | 15.52 | 25 | 60 | 15 | 0 | 3142 | 45 |
| WES26 | 7.51 | 869 | 19.95 | 1.0 | 3.07 | 0.04 | 10.75 | 42.98 | 20 | 70 | 10 | 0 | 1257 | 55 |
| WES27 | 8.19 | 1437 | 23.02 | 2.0 | 7.33 | 0.04 | 1.69 | 5.61 | 60 | 40 | 0 | 0 | 17868 | 45 |
| WES28 | 8.23 | 890 | 22.00 | 2.0 | 7.33 | 0.05 | 0.00 | 10.64 | 30 | 50 | 20 | 0 | 3000 | 64 |
|  |  |  |  |  |  |  |  |  |  |  |  |  |  |  |

**Appendix S4.** Summary statistics of the physico-chemical variables (untransformed data) collected in this study, reported per wetland cluster (defined by terrestrial vegetation group). EC – electrical conductivity

| Ferricrete fynbos (n = 6) | pH | EC (µS.cm^-1^) | Ave temp. (°C) | Turbidity (NTU) | Dissolved oxygen (mg.L^-1^) | NO_3_^-^ + NO_2_^-^ (μg.L^-1^) | PO_4_^3+^ (μg.L^-1^) | NH_4_^+^ (μg.L^-1^) |
| --- | --- | --- | --- | --- | --- | --- | --- | --- |
| Mean | 8.30 | 4382 | 21.9 | 5.7 | 9.53 | 1.76 | 7.13 | 17.87 |
| Standard deviation | 1.13 | 6667 | 3.6 | 4.1 | 2.02 | 2.61 | 6.61 | 5.26 |
| Median | 8.31 | 1809 | 21.4 | 3.8 | 9.85 | 0.93 | 3.90 | 18.59 |
| 25 percentile | 7.44 | 1066 | 19.3 | 3.1 | 8.30 | 0.00 | 3.47 | 16.61 |
| 75 percentile | 8.73 | 2816 | 22.3 | 8.1 | 10.28 | 1.96 | 9.43 | 21.24 |
| Minimum | 7.05 | 825 | 18.8 | 2.0 | 6.77 | 0.00 | 1.58 | 8.57 |
| Maximum | 10.12 | 17880 | 28.6 | 12.0 | 12.53 | 6.73 | 18.80 | 23.40 |
| Sand fynbos (n = 44) | | | | | | | | |
| Mean | 7.28 | 3102 | 17.5 | 5.6 | 6.41 | 225.47 | 254.54 | 296.55 |
| Standard deviation | 1.29 | 4918 | 4.0 | 6.6 | 3.50 | 1245.43 | 516.20 | 810.87 |
| Median | 7.43 | 581 | 16.9 | 3.4 | 5.82 | 4.17 | 35.82 | 33.34 |
| 25 percentile | 6.77 | 253 | 15.0 | 1.7 | 3.99 | 0.98 | 5.19 | 19.59 |
| 75 percentile | 8.21 | 3409 | 21.1 | 5.6 | 7.85 | 10.34 | 224.44 | 65.44 |
| Minimum | 4.07 | 114 | 11.3 | 0.7 | 1.20 | 0.01 | 1.50 | 6.70 |
| Maximum | 9.69 | 20833 | 26.3 | 34.0 | 16.70 | 8241.59 | 2827.36 | 4231.53 |
| Sandstone fynbos (n = 6) | | | | | | | | |
| Mean | 8.06 | 3943 | 20.8 | 4.2 | 8.74 | 8.58 | 7.64 | 38.43 |
| Standard deviation | 0.99 | 4760 | 4.4 | 4.3 | 1.23 | 8.42 | 3.00 | 23.37 |
| Median | 7.92 | 2673 | 22.1 | 2.5 | 8.85 | 7.22 | 7.89 | 39.67 |
| 25 percentile | 7.75 | 1303 | 17.1 | 1.8 | 8.68 | 1.38 | 6.34 | 36.32 |
| 75 percentile | 8.26 | 3017 | 24.5 | 4.4 | 9.25 | 16.10 | 9.57 | 41.47 |
| Minimum | 6.71 | 876 | 15.0 | 1.0 | 6.53 | 0.00 | 2.94 | 0.23 |
| Maximum | 9.74 | 13460 | 24.9 | 12.5 | 10.23 | 18.54 | 11.16 | 73.75 |
| Shale renosterveld (n = 6) | | | | | | | | |
| Mean | 7.30 | 359 | 17.0 | 235.3 | 4.62 | 12.73 | 301.75 | 798.87 |
| Standard deviation | 0.25 | 91 | 2.5 | 282.4 | 1.29 | 17.80 | 403.01 | 1135.28 |
| Median | 7.29 | 354 | 17.4 | 105.7 | 4.05 | 5.78 | 101.81 | 147.45 |
| 25 percentile | 7.23 | 287 | 17.0 | 42.0 | 3.86 | 3.33 | 28.46 | 106.49 |
| 75 percentile | 7.34 | 415 | 18.1 | 369.0 | 5.44 | 12.41 | 469.01 | 1191.80 |
| Minimum | 6.95 | 260 | 12.3 | 9.8 | 3.33 | 0.06 | 12.03 | 61.72 |
| Maximum | 7.72 | 488 | 19.7 | 713.0 | 6.57 | 47.65 | 999.41 | 2803.87 |
| Western strandveld (n = 28) | | | | | | | | |
| Mean | 8.04 | 3178 | 18.2 | 4.2 | 7.06 | 6.38 | 19.17 | 35.23 |
| Standard deviation | 0.37 | 2819 | 3.6 | 8.3 | 2.63 | 15.17 | 33.27 | 26.48 |
| Median | 8.12 | 2057 | 18.3 | 1.7 | 7.20 | 1.60 | 7.84 | 32.84 |
| 25 percentile | 7.77 | 1134 | 15.6 | 1.1 | 5.61 | 0.67 | 4.20 | 15.77 |
| 75 percentile | 8.23 | 3858 | 22.1 | 3.2 | 8.44 | 2.86 | 14.78 | 44.82 |
| Minimum | 7.33 | 840 | 11.2 | 0.4 | 2.00 | 0.04 | 0.00 | 1.47 |
| Maximum | 9.01 | 10640 | 23.7 | 44.2 | 13.33 | 60.67 | 166.28 | 109.57 |
|  |  |  |  |  |  |  |  |  |
